# Impact of domestic white LED light on cognitive functions and amelioration of blue light blocking lens (BBL) on healthy adults

**DOI:** 10.1016/j.nbscr.2025.100119

**Published:** 2025-04-17

**Authors:** Mousumi Ghosh, Hari Prakash Palaniswamy, Nishitha. G, Stelyna Joylin, Shwetha. T.S, M. Sanjana, R.P. Radhika, Nagarajan Theruveethi

**Affiliations:** a Department of Optometry, Manipal College of Health Professions, Manipal Academy of Higher Education, Manipal, 576104, India; b Department of Speech and Hearing, Manipal College of Health Professions, Manipal Academy of Higher Education, Manipal, 576104, India; c Department of Clinical Psychology, Manipal College of Health Professions, Manipal Academy of Higher Education, Manipal, 576104, India; d Department of Optometry, Dr B.C.Roy Academy of Professional Courses, Durgapur, West Bengal, India; e Department of Optometry, Srinivas Institute of Allied Health Sciences, Srinivas University, Mangalore, India

**Keywords:** Light-emitting diode, Intrinsically photosensitive retinal ganglion cells, Cognition, Blue-blocking lens, Event-related potential

## Abstract

White light-emitting diodes **(**WLEDs) can affect cognition and working memory. Blue light-blocking lenses (BBL) may help alleviate this. We aim to study the relationship between WLED and the ameliorative effect of BBL. We included 15 healthy participants based on the PSQI and Mini-Cog™ screening. The eligible participants underwent a baseline recording of event-related potential (ERP) of P300 using electroencephalography (EEG) while performing a 2-back task, followed by exposure to WLED (600 lux) that was given (45° with 80 cm apart from the participant's eye plane) for 30 min. A similar protocol was maintained when BBL was worn with WLED exposure. The participants' mean PSQI and Mini-Cog™ scores (n = 15) were 3 and 5, respectively. The behavioral functioning of participants using a 2-back task revealed enhancement in working memory cognition by fastening the response time (ms) from base to post-WLED to post-WLED + BBL (p < 0.001). Still, no significant difference (p > 0.05) in accuracy (%) was observed. The learning effect in the control group using a 2-back task revealed no statistically significant difference (p > 0.05) in both accuracy (%) and response time (ms). Additionally, no significant change (p > 0.05) was found within the three light groups in latency (ms) and amplitude (μV) at the P300 region of ERP in the prefrontal cortex. The existing results found that domestic WLED exposure significantly leads to a faster response time in working memory performance in the prefrontal cortex, thus remaining alert. BBL is not protective in the nonvisual senses when exposed to WLED for 30 min.

## Highlights

• Research has shown that white LED lights emit blue light, which has been found to have a stimulating effect on the brain. This can alter memory and increase alertness.
• Blue light exposure during nighttime can activate the photoentrainment pathway and non-visual senses.
• Cumulative exposure to WLED has improved the non-visual senses' alertness in the prefrontal cortex.
• BBL doesn’t have a protective role against blue light (WLED) exposure; it triggers alertness and leads to phase delay.

## 1 Introduction

Electric lighting (EL) has become essential to modern life, providing light and increasing productivity (Françon et al., 2024). The over-reliance on electric lighting has been causing a significant increase in energy consumption and excessive emissions of CO_2_ (Behar-Cohen et al., 2011a). The world is estimated to consume approximately 2650 billion megawatt-hours of electricity annually to power electric lighting, constituting nearly 19 % of global electricity production and contributing to a more energy-sustainable future (Sena et al., 2024). The advancement of EL and light-emitting diode (LED) technology has revolutionised the electricity market, offering soothing and energising illumination to mitigate environmental risks and foster eco-friendliness, in contrast to traditional lamps (Campbell, 2023). LEDs are increasingly gaining prominence in domestic lighting due to their spectral attributes and energy efficiency (Sena et al., 2024). This entails the fusion of a blue LED with yellow phosphors by emitting photons of longer wavelengths, thus yielding a white appearance (Campbell, 2023). However, the transition to LED lighting has meant a resurgence in exposure to blue wavelengths (Campbell, 2023). This dynamic property promises to improve cognitive performance, mood regulation, sleep patterns, and overall well-being. CIE standards globally have embraced LED lighting policies by introducing LED-integrated products to enhance health, mood, or well-being, often without robust scientific evidence to support their claims (Houser et al., 2021).

The neurosensory retinal (human) system of classical photoreceptors (visual) cone and rod cells to distinguish light from dark and non-classical photoreceptors (non-visual) sensory functions for the circadian system the intrinsically photosensitive retinal ganglion cells (ipRGCs) dedicated to regulating behaviour and physiology due to its presence of photopigment-melanopsin (Lucas et al., 2014; Wässle, 2004). Impact on these systems depends on the timing and intensity of light exposure (Bedrosian and Nelson, 2017). This implies that increased exposure to blue-wavelength light (∼460–480 nm) can have positive and negative impacts (Gaston et al., 2015).

The monochromatic blue spectrum can adversely affect visual perception and influence non-visual sensory functions mediated by ipRGCs. These cells, primarily sensitive to light but pivotal for non-visual processes such as pupillary reflexes and circadian rhythm regulation, are affected by the shorter wavelengths of blue light (Elenberger et al., 2020). Such exposure alters circadian rhythms by reducing melatonin production, cortisol levels, cognitive performance, mood, and alertness (Alkozei et al., 2016a, 2017; Blume et al., 2019a). The suprachiasmatic nucleus (SCN), acting as the master clock, receives input from ipRGCs, synchronising non-visual functions based on ambient light levels (Blume et al., 2019b; Alkozei et al., 2016b). Cumulative exposure to blue light suppresses melatonin secretion, prolonging the sleep cycle (Alkozei et al., 2016a, 2017).

The rodent studies have documented potential risks to retinal health posed by the spectral composition of WLEDs with an intensity of 1000 lux or more, mainly due to blue light spanning approximately 400–490 nm (Shang et al., 2014). This high-energy blue light has been associated with denaturing crucial retinal proteins, generating free radicals, and potentially compromising retinal tissue integrity, leading to retinal apoptosis (Behar-Cohen et al., 2011b; Youssef et al., 2011a; Wu et al., 2006). Prolonged exposure to WLEDs or concentrated light sources is commonly associated with the potential hazards of blue light (Menéndez-Velázquez et al., 2022; Dauchy et al., 2016). Research indicates that low-energy light sources pose minimal risk of blue light hazard (BLH) to the retina (Grimm et al.), circadian system (Blume et al., 2019b; Alkozei et al., 2016b), metabolic function (Cheung et al., 2016) and cognitive functions (Chellappa et al., 2011a). However, the ipRGCs are linked to photophobia in migraines and exposure to blue light (McAdams et al., 2020). Despite the negative connotations, blue-enriched light can enhance the sleep quality and mood of Alzheimer's patients (Kim et al., 2021) by adjusting their light spectrum and intensity (Houser et al., 2021). Moreover, blue light exposure influences brain regions of the hypothalamus and the locus coeruleus (LC), releasing norepinephrine and enhancing alertness and cognitive functions (Alkozei et al., 2016a, 2017). Studies have shown increased activation within the dorsolateral and ventrolateral prefrontal cortex (DLPFC and VLPFC) in response to blue light exposure, indicating enhanced cognitive processing and working memory in nondeprived individuals (Alkozei et al., 2016a). To mitigate the adverse effects of blue light exposure, blue-blocking lenses (BBLs) have been developed to selectively filter out harmful shorter wavelengths, reducing phototoxicity, regulating circadian rhythms, and improving sleep quality (Alzahrani et al., 2019, 2020). These lenses can alleviate eye strain, dryness, and fatigue associated with prolonged LED screen use and enhance sleep quality (Downie et al., 2019). The studies have not explored the potential of BBLs on cognition and behavior despite being marketised to improve non-visual senses.

LED light has environmental and human health advantages, including visual and non-visual performance, like cognition, sleep intensity, and day-stretch alertness (Bedrosian and Nelson, 2017). The active engagement between the human body and indoor lighting demands more than merely a circadian clock. It also necessitates IpRGC pathways that transport the environmental information to the SCN for excellent circadian entrainment; additionally, output pathways are essential for transmitting timing information back to the body, ensuring that bodily functions are synchronised (Blume et al., 2019b).

Counting this, cumulative exposure to LED light could delay sleep when exposed beyond working hours by suppressing melatonin and altering cognition by collapsing the circadian cycle. This study investigates the cognitive effects and working memory response on the prefrontal cortex after post-WLED exposure and the amelioration of commercially available BBLs (Crizal Prevencia) against cumulative WLED exposure in healthy adults.

## 2 Materials and methods

**Ethical Statement:** This experimental crossover study was conducted in the Cognitive Research Laboratory of MCHP, MAHE, Manipal, India. After obtaining Ethics approval (IEC1:135/2022), (CTRI No: CTRI/2022/09/045603, dated 20-Sep-2022).

**Participant Recruitment:** We included 15 healthy participants, consisting of four males (with a mean age of 22.00 ± 1.633 years) and 11 females (with a mean age of 23.09 ± 2.548 years). Written consent was obtained from all participants after adhering to the COVID-19 protocol. These participants were proficient in English, had a regular sleep schedule of 7–8 h (bedtime: 10:00 p.m.-11:00 p.m.; wakeup time: 6:00 a.m.-7:00 a.m., Indian standard time - IST), had a global Pittsburgh Sleep Quality Index (PSQI) score of ≤5 (below the threshold for sleep disturbance) (Farah et al., 2019), and a Mini-Cog™ score of >3 (below threshold cognitive impairment) (Borson et al., 2003). Additionally, they had a corrected distance visual acuity (V/A) better than or equal to 0.1 log MAR with a healthy retina. Participants with any ocular pathology, psychological and neurological disorders, intake of systemic medications, smoking, alcoholism, consumption of more than two cups of coffee per day (within a 24-h cycle)/more than 240 mg of caffeine per day (Beaven and Ekström, 2013), along with any systemic illness, were excluded from the study.

**Fig. 1:**
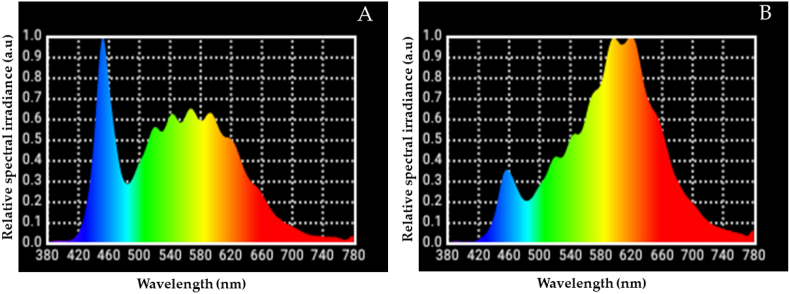
**1A:** Spectral power distribution (SPD) of WLED, x-axis: wavelength (nm) and y-axis: relative spectral irradiance (arbitrary unit); **1B:** SPD of Amber LED, x-axis: wavelength (nm) and y-axis: relative spectral irradiance (arbitrary unit) measured using a light spectrometer.

**Light parameters standardisation:** The study experiment was conducted to standardise the lighting setup at the Department of Electrical and Electronics Engineering, MIT lighting laboratory. The group that was exposed to light included a polychromatic white LED (Philips Ace Saver LED Lamp 4 W E 27, 600 lux, Philips India Pvt. Ltd.) and a monochromatic amber LED (Panasonic LED Bright & Energy Saving Bulb 5-W, 470 lux, Panasonic India Pvt. Ltd.). The two LEDs' light parameters were measured using a light spectrometer (Asensetek). The correlated colour temperature (CCT) of the WLED and Amber LED was 5461 K and 2741 K, respectively. The spectral power distribution of the white and amber LEDs was measured using a light spectrometer (Fig. 1A and B).

To calculate the retinal irradiance of the participants according to their pupil diameter and the area of the retina exposed, the following formula was used: (Eret = Ecor ∗ (πD2/4) ∗τ/Aret) (Youssef et al., 2011b). Here, Eret is the retinal irradiance in W/cm^2^, Ecor is the corneal irradiance in W/cm^2^, D is the diameter of the pupil in mm, Aret is the area of the irradiated retina in mm^2^, and τ is the ocular media transmission in nm. Before the experiment, the right eye of each participant was dilated with ITROP PLUS Eye Drops (tropicamide 0.8 %, phenylephrine 5 %, Mfg. by Cipla Ltd, India) to maintain uniform retinal illuminance. The average pupil diameter of the participants is 7 mm, and the retinal irradiance value after calculation is 0.0169 W/cm^2^. The safe retinal irradiance value is 12.7 W/cm^2^, and the equivalent safe corneal irradiance for a subtended angle of 9.3 mrad is 0.16 W/cm^2^ or 1600 W/m^2^—the transmission coefficient, τ (∼0.5).

## 3 Experimental procedure

### 3.1 Screening

The study included participants who scored = 5 on the global PSQI score and >3 on the Mini-Cog™ test. All participants were screened using the PSQI and Mini-Cog™ testing to ensure the study's accuracy. Those who met the criteria, scoring = 5 (below threshold sleep disturbance) on the global PSQI score (Table 1) and >3 (below threshold cognitive impairment) on the Mini-Cog™, were included in the study. This method was implemented to maintain a fair and consistent standard for all participants.

**Table 1:** Descriptive statistics.

Descriptive statistics	WLED n = 10, Controls n = 5
Mean age in years (Mean ± S.D)	Female = 22.29 ± 2.430, Female = 24.5 ± 2.38
Gender ratio (Male: Female)	1:3
Mean Global PSQI score	3 (good category)
Average hours of sleep per night (Mean ± S.D)	7.53 ± 0.5
Average bedtime (Mean ± S.D)	11.52 ± 0.423
Average wake-up time (Mean ± S.D)	7.05 ± 0.29
Mean Mini-Cog™ score	5 (normal)

### 3.2 Electroencephalography (EEG) electrode preparation

**Fig. 2:**
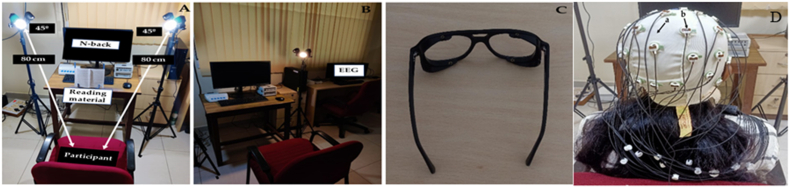
**Light exposure setup with EEG measurements.** 2**A:** White LED (600 lux each) exposure set up along with the distance between the participant's eye and white light is approximately 80 cm placed at a 45-degree angle. **2B:** Amber LED (470 lux each) exposure setup required for washout of white light. **2C:** Customizable wrap-around blue-blocking lens (BBL). **2D:** Electroencephalography (EEG) Electrocap on the participant's scalp with 32 electrodes, (a) EEG scalp cap; (b) EEG electrodes.

The participants were prepared for EEG recordings by following the standard procedure. They wore an EEG Electrocap with 32 electrodes (actiCAP slim) attached to their scalp (Fig. 2 D). Before wearing the cap, the forehead skin was cleaned using a skin-preparing agent (NuPrep) and a cotton swab to remove dirt or oil from the skin, ensuring precise impedance levels. Each electrode was then filled with a conducting gel (SuperVisc-1000 gr.) using a blunted needle, which ensured an excellent EEG impedance level (10–20 kOhm). The electrodes were connected to an EEG amplifier (BrainVision actiCHamp PLUS) that converted amplified filtered signals from analogue to digital signals. These signals were transmitted to the recording computer (Lenovo D, Hong Fu Jin Precision Electronics, Chongqing Co. Ltd.) for further processing.

### 3.3 Event-related potential (ERP)

During the N-back (2-back) working memory task, we recorded EEG to measure participants' cognitive function in the prefrontal cortex. We then extracted ERP recordings of 2 negative and three positive waveforms: N1, N2, P1, P2, and P3. These waveforms were characterised by their latency and amplitude. We used P3 to evaluate cognitive performance. The latency of the P3 wave indicated the speed of discrimination between events, measured in milliseconds (ms). The amplitude of the P3 wave represented attention to stimuli information, measured in microvolts (μV). A shorter latency peak indicates better mental performance, while a higher amplitude peak suggests better attention.

### 3.4 N-back task

To assess the working memory function of a participant by keeping them engaged with the task as a cognitive task that includes cognitive load with four conditions, i.e., “0-back” to “3-back” loads. We used the N-back task to present a series of stimuli such as letters, numbers, or pictures, and participants must recall and match the letter displayed with the one presented n-items ago, where n is a variable (Hunsley and Allan, 2019). The participants 2-back working memory task (E-Studio, E-Prime® 3.0 Build 3.0.3.214 ©1996–2022 Psychology Software Tools, Inc. Pittsburgh, USA) was given to the participants using a number as a stimulus or target and had to recall and match the target projected two trials back. Participants responded to each stimulus by pressing the Chronos response box keyboard (Chronos®, Psychology Software Tools, Inc. Pittsburgh, USA) using the right-hand index finger for targeted stimuli and the left-hand index finger for nontargeted stimuli.

### 3.5 Blue-blocking lens

We used the Crizal® Prevencia™ lens (Essilor), and the lenses were fitted with specialised wrap-around explicit frame material to ensure the light will not enter other than a specified area. Additionally, it has been verified to attenuate blue-violet rays by 17.80 % (Alzahrani et al., 2019) and boasts an Eye-Sun Protection Factor (E-SPF®) of 25.

### 3.6 Light exposure setup

During the experimental procedure, two light setups were used - a white LED exposure setup and an amber LED exposure setup. The white LED exposure setup (Fig. 2A) consisted of two white LEDs, each emitting 600 lux, placed at both sides of each participant at an angle of 45° and 80 cm from their eye. Similarly, the amber LED exposure setup (as seen in Fig. 2B) used two amber LEDs, each emitting 470 lux, placed at both sides of each participant at an angle of approximately 45° and 80 cm from their eye (Alkozei et al., 2016b). To carry out the experimental procedure, a customised wrap-around blue-blocking lens (BBL) from Crizal® Prevencia™, Essilor France, was used, as shown in Fig. 2C.

### 3.7 Procedure

A detailed explanation of the procedure, including a practice session on the 2-back paradigm, was provided to the participants. The study experiment consisted of two methods. In the first procedure, 10 out of 15 participants had their right eye dilated using medication while viewing through both eyes. EEG recordings were taken during eyes open (EO) and eyes closed (EC) resting state procedures, each lasting 2 min. After that, baseline brain activity was measured while participants engaged in a working memory task using a (N-back) 2-back paradigm. ERP recordings were also taken for 10 min in a darkened room without light exposure. Participants showed no decline in accommodative ability during the task, as optotypes were N60 and printed in transparent, legible black. Participants also demonstrated a visual acuity (V/A) equal to or better than 0.1 log MAR, indicating a healthy retina. Following this, participants were exposed to WLEDs for 20 min while EEG recordings were conducted during EO and EC resting states. Lights were approximately 45° and 80 cm from the participant's eyes. To maintain task engagement, participants were asked to read moral stories from a printed book (Moral Stories, written by Er. B.G. Ramesh and published by Ganesh Publications, Balepet, Bangalore-53, Karnataka). The book's font size was N10 in black print, and the stories were chosen to ensure they did not affect participants' moods.

After the WLED exposure, participants experienced a washout period of 30 min with amber LEDs. During this time, participants read the book in a darkened room, and no working memory tasks or ERP recordings were conducted. Subsequently, EEG recordings during EO and EC resting states were performed, followed by another 20-min exposure to WLEDs while wearing BBL (Crizal Prevencia). Participants continued reading the book during this period, and a 10-min session of the 2-back working memory task was conducted with WLED exposure while wearing BBL, accompanied by ERP recordings in the darkened room.

**Fig. 3:**
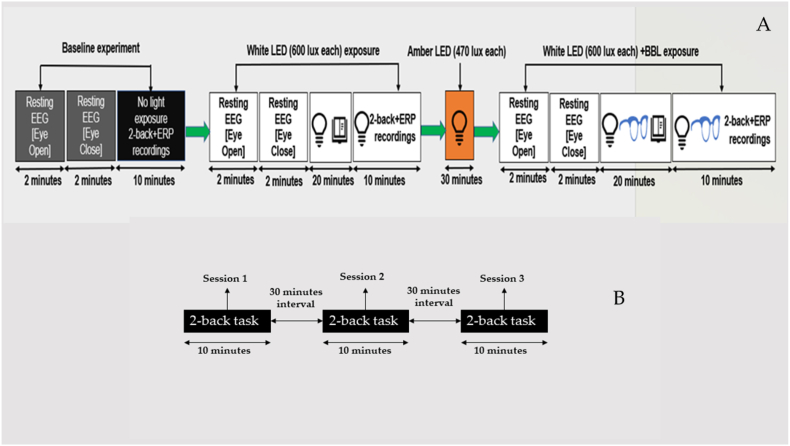
Schematic representation of the **3A**: Light exposure and **3B:** without light exposure.

The second procedure involved the remaining five participants who served as controls. This procedure consisted of three sessions where participants performed the 2-back working memory task without WLED or other light exposure. Each session was followed by a 30-min interval during which participants were instructed to relax and refrain from doing any activities. After completing the entire procedure, the results were recorded and analysed. The schematic representations of the two methods are depicted in Fig. 3A and B, respectively.

### 3.8 EEG and ERP data collection

The EEG data was filtered using MATLAB (version R2022b) and EEGLAB software. The channel data scroll was selected to remove noisy segments, and the reject option was clicked. Similarly, the ERP data underwent cleaning procedures in the same software, involving decomposition by ICA and removing components. The data was collected at a frequency of 600 Hz.

### 3.9 Statistical analysis

Data was analysed using IBM SPSS Statistics Version 20.0 and MATLAB. Kolmogorov-Smirnov test (KS) determined normality and statistical significance at p < 0.05. Ten healthy participants (3 males and 7 females) were exposed to WLED, with a control group of 5 healthy participants. Participants slept an average of 7.53 h per night and had a mean Mini-Cog™ score of 5.

### 3.10 Behavioural analysis

**Fig. 4:**
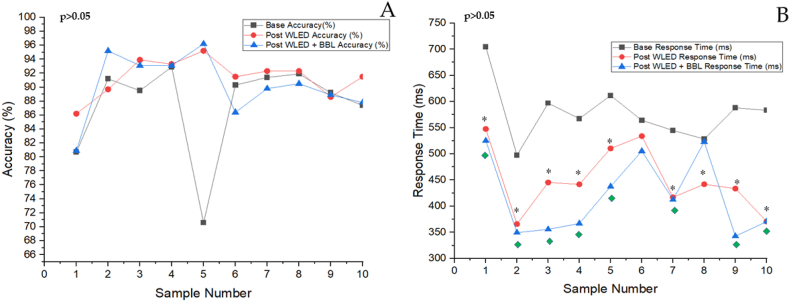
**Behavioral functioning in participants using a 2-back task with WLED exposure.** The line graph describes the Y-axis **4A:** accuracy (%) and **4B:** response time (ms) for the 2-back task against the X-axis: each participant (sample number), where base, post-WLED and post-WLED + BBL for both accuracy (%) and response time (ms). *p values* represent the significance of the repeated-measures ANOVA within each light group. A significant difference (p < 0.001) between pairs using Bonferroni correction in response time (ms) is indicated by the following symbols: ∗ base response time vs post-WLED response time;  base response time vs post-WLED + BBL response time.

Ten healthy participants were involved in the first experimental procedure. The normality of the behavioral data K-S is usually distributed with a significance value (p > 0.05). Therefore, Repeated-ANOVA with Bonferroni correction was conducted to evaluate the accuracy (%) and response time (ms) of the participants when performing the 2-back task between different lighting groups (base and post-WLED), (base and post-WLED + BBL), and (post-WLED and post-WLED + BBL). The results showed no statistically significant difference in accuracy (%) for the 2-back task between the groups (base and post-WLED, p = 0.408), (base and post-WLED + BBL, p > 0.999), and (post-WLED and post-WLED + BBL, p = 0.742) (Fig. 4A). However, a significant difference was found in response time (ms) for the 2-back task between the groups (base and post-WLED, p < 0.001) (base and post-WLED + BBL, p < 0.001) (Fig. 4B). Moreover, there was no statistically significant difference in response time (ms) for the 2-back task between groups (post-WLED and post-WLED + BBL, p = 0.270).

**Fig. 5:**
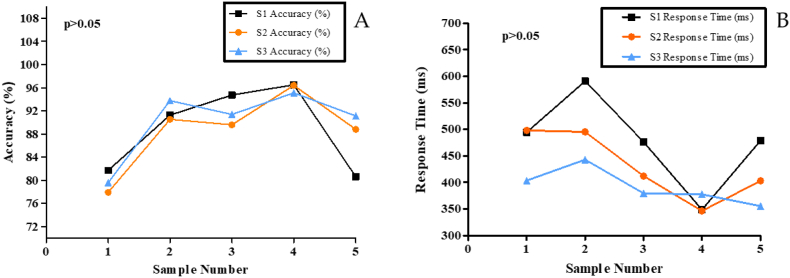
**Learning effect in participants using the 2-back task for three sessions without WLED exposure.** The line graph describes the Y-axis **5A:** accuracy (%) and **5B:** response time (ms) for the 2-back task against the X-axis: each participant (sample number), where S1, S2, and S3 for both accuracy (%) and response time (ms). *p values* represent the significance of the Related-Samples Friedman's Two-Way Analysis of Variance by Rank test within each 2-back task session.

The learning effect assessed on five participants A related-sample Friedman's two-way analysis of variance by rank test was performed to evaluate the accuracy (%) and response time (ms) of the participants within the three sessions (S1, S2, S3) of the 2-back task. The results showed no statistically significant difference (p = 0.247) in accuracy (%) for the 2-back task within the three sessions (Fig. 5A). Similarly, no significant differences (p = 0.165) were found in response time (ms) for the same (Fig. 5B).

### 3.11 Prefrontal cortical response in ERP (P300)

**Fig. 6:**
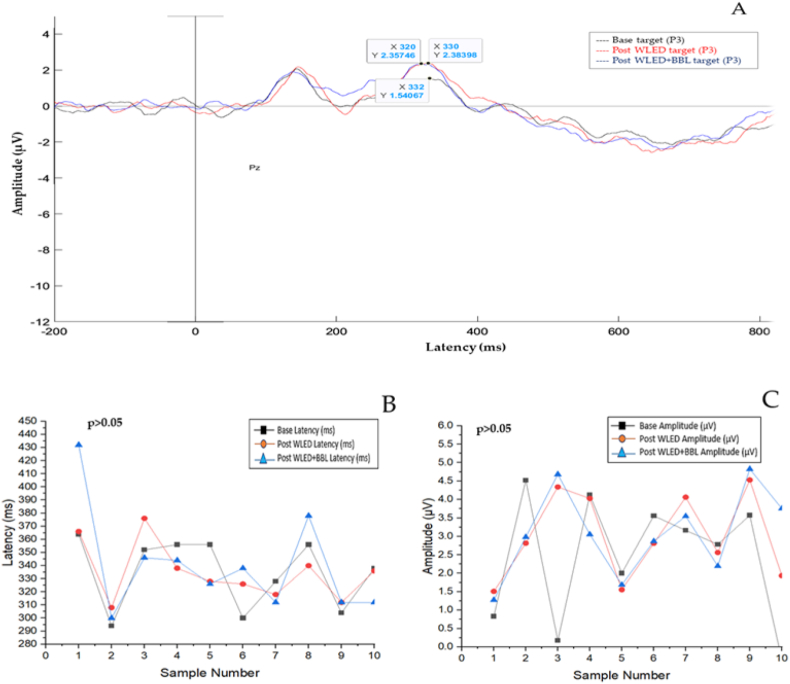
Prefrontal cortical response in participants with WLED exposure using ERP at P300**. 6A:** Grand average ERP (P300) values of x-axis: latency (ms) against y-axis: amplitude (μV) for each light group-base target represented in black line, post WLED target in red line and post WLED + BBL in blue line assessed using MATLAB EEGLAB. The line graph describes the y-axis **6B:** latency (ms) and **6C:** amplitude (μV) for the 2-back task against the x-axis: each participant (sample number), where base, post-WLED and post-WLED + BBL for both latency (ms) and amplitude (μV). *p values* represent the significance of the repeated-measures ANOVA within each light group.

The cognitive potential of 10 participants was analysed using the Event-Related Potential (ERP) in the prefrontal cortex, specifically at the P300 region, with MATLAB EEGLAB (version R2022b). We measured the change in latency (ms) and amplitude (μV) using the x-axis and y-axis, respectively. The results showed no marked changes in the base, post-WLED, and post-WLED + BBL groups. Table 2 and Fig. 6A provide detailed results on latency (ms) [332, 330, and 320] and amplitude (μV) [1.54067, 2.38398, and 2.35746].

**Table 2:** Grand average ERP (P300) values of amplitude (μV) and latency (ms).

Grand average ERP (P300)	X-axis: Latency (ms)	Y-axis: Amplitude (μV)
Base target	332	1.54067
Post WLED target	330	2.38398
Post WLED + BBL target	320	2.35746

Furthermore, the study used repeated-measures ANOVA with Bonferroni correction to examine the ERP's latency (ms) and amplitude (μV) at P300 in the prefrontal cortex. At the same time, the participants performed a working memory task. The study did not find any statistically significant differences (p > 0.05) between the lighting groups (base and post-WLED), (base and post-WLED + BBL), and (post-WLED and post-WLED + BBL). Fig. 6B and C provide a graphical representation of the results.

## 4 Discussion

We observed a significant decrease in response time (ms) from the baseline to post-WLED and post-WLED + BBL phases, indicating improved working memory performance speed after exposure to WLED. This could be due to the stimulation of ipRGCs, which might have contributed to an increase in baseline activation of the LC and may have played a role in the release of norepinephrine throughout the prefrontal cortex. This reaction enhanced alertness and improved the brain's performance (Alkozei et al., 2016a).

In the behavioral N-back task for both response time (RT) and accuracy, significant differences were found in RT; as the difficulty of the N-back task was increased, i.e., from the 0-back to 2-back condition, the RT also increased (Kossowski et al., 2021). Blue light can enhance cognition and cortical function, which may persist for a shorter duration (up to 40 min) post-cessation of the light (Alkozei et al., 2016b). WLEDs emit blue light at their peak, which has elicited a similar response in our research, as brief exposure to blue light can enhance neural activation in the prefrontal regions crucial for working memory (Alkozei et al., 2016b). In a recent study, it was observed that the response for the 0-back condition was faster compared to the 1-back and 2-back conditions for all wavelengths of light, including ambient light, blue light (peaking at λ = 470 nm), green light (peaking at λ = 505 nm), and red light (peaking at λ = 630 nm). This finding supports the notion that exposure to blue light for approximately 30 min may enhance working memory, as has been addressed in previous studies (Alkozei et al., 2016a). It can either synchronise or phase-shift the circadian rhythm or immediately improve the performance in tasks like digit recall, serial addition and subtraction, and simple reaction time tasks through non-visual performance through ipRGCs and exploits the physiological rhythm (Chellappa et al., 2011b; Vandewalle et al., 2006). It evokes systemic melatonin rejoinders and alters behaviour and cognitive performance (Cajochen et al., 2011). There was no significant difference in the accuracy of behavioural responses while performing the working memory task with a 2-back condition across all light groups (base to post-WLED to post-WLED + BBL). These results are consistent with Anna Alkozei and colleagues' study in 2016, which also showed no group difference in accuracy following prolonged cumulative short-wavelength light exposure.

The temporary impact of light exposure on the brain may cause this effect as the subcortical regions are activated more quickly than cortical regions. Still, their activity declines as the cortex requires more intense and prolonged stimulation. Research has shown that even an exposure of 20 min to bright white light can significantly impact the brain's activity. They found that the subcortical regions of the brain responded more quickly to light exposure, causing changes in thalamic and cortical activity. Although these changes gradually subsided after the light exposure, they were still long-lasting and positively impacted the overall brain function (Chellappa et al., 2011a).

No significant changes were observed in working memory and cognitive performance in WLED + BBL treatment. Few recent research studies have indicated that using BBLs may not provide substantial relief for digital eye strain in individuals who do not exhibit symptoms of eye fatigue (Singh et al., 2021) and sleep quality (Singh et al., 2023). However, observations support that BBLs reduce computer vision syndrome (Alzahran et al., 2021) and enhance sleep quality in nulliparous pregnant women (Liset et al., 2022). There is conflicting evidence regarding BBLs' ability to strengthen non-visual senses. This can make it challenging for practitioners and consumers to make informed decisions. Therefore, it is important to continue exploring the potential benefits of BBLs while also considering their potential risks and limitations.

To strengthen our current work, we analysed the learning effects of a control group through small samples. We aimed to assess their accuracy (%) and response time (ms) while performing a 2-back working memory task for three sessions. There was a 30-min interval between each session. We observed no statistically significant difference in accuracy (%) between each session with the 2-back task in the control group. This aligns with our study's behavioural analysis of the intervention group with WLED exposure. However, unlike the intervention group, we observed no decrease in the response time (ms) for each session with the 2-back task in the control group. This indicates no faster response in the working memory task with increased sessions. Therefore, alertness is not enhanced without WLED exposure. No significant change in either the latency (ms) or amplitude (μV) of cognitive functioning was observed in the prefrontal cortex at the P300 region in ERP, which innervates through the ipRGCs when exposed to WLED for 30 min. However, exposure to blue light for 30 min resulted in better memory retention after about 1.5 h of memory consolidation due to the activation of the LC(11).

Our findings and previous research suggest that intrinsically photosensitive retinal ganglion cells (ipRGCs) in humans play a double role in non-visual senses. These cells are responsible for physiological and behavioural responses during prolonged exposure to polychromatic white light for about 30 min. The signals are transmitted through the SCN in the hypothalamus and other brain regions, such as the prefrontal cortex, and the locus coeruleus (LC) is activated by releasing norepinephrine. Activation of melanopsin in ipRGCs can sustain light signals for up to 40 min after light stimulation has ceased. This opens new possibilities for exploring the human body's response to light and its impact on overall health and well-being (Mai-Yi Fan et al., 2018).

Alterations in functional nonvisual senses lead to phase delay in circadian rhythm, and keeping alert due to shorter wavelength exposure in the evening results in delayed morning wakefulness (Alkozei et al., 2016b). This alteration occurs due to the suppression of melatonin secretion when melanopsin in ipRGCs absorbs the blue wavelength (approximately 480 nm) (Men et al., 2022). Melatonin secretion peaks during the night, with the highest circadian drive for sleep occurring between 2 and 6 a.m. (Cajochen, 2007). Commercially available BBLs have been advertised as protecting glasses for individuals using computers and other electronic devices in the evening by maintaining the circadian photoentrainment pathway (Alzahrani et al., 2020). Studies have theoretically shown that BBLs fade the transmitted shorter wavelength blue, reducing blue perception and ipRGC sensitivities (Alzahrani et al., 2020). In the present study, post-WLED exposure for 30 min with BBL (Crizal Prevencia) did not show any ameliorative changes assessed in ERP (P300) at the prefrontal cortical region.

We used iridescent WLED exposure on the same day with different measurements that might have influenced the working memory performance in the prefrontal cortex, such as alertness. Thus, the present study findings may not be directly comparable with studies using objectively measured light-induced cognitive performances. The authors experimented with a smaller sample size, which limits the generalizability of the findings and reduces the statistical power. also, they did not experiment in a particular period, such as evening time, and assessed for the phase delay in circadian rhythm. Although the screening of the sleep quality or the sustained cognitive effects of the participants was done before the experiment using the PSQI scoring and post-WLED exposure, the sleep quality assessment was not done for them, which is necessary to understand the full impact of the WLED and BBL exposure. Additionally, studies that need to be explored on mapping biomarkers with ipRGCs are the few limitations of our study.

## 5 Limitations and future directions

While the current study provides valuable insights into the effects of WLED exposure on working memory and the role of BBLs, it has certain limitations. The small sample size (n = 15) may limit the generalizability of the findings and reduce statistical power, particularly for subtle neural effects like P300 amplitude and latency changes. Future studies can include more populations varying with age and other characteristics. Additionally, the study focused solely on the prefrontal cortex; future research could explore other brain regions (e.g., parietal cortex or hippocampus) to map blue light's cognitive impacts comprehensively. The lack of follow-up measures precludes conclusions about long-term or sustained effects of WLED exposure, such as circadian disruption or sleep quality alterations. Furthermore, the experiment was conducted during the daytime, which may not capture the full influence of blue light on evening alertness or melatonin suppression. Future studies should employ larger cohorts, longitudinal designs, and broader neuroimaging techniques (e.g., fMRI) to validate these findings. Investigating the efficacy of BBLs under varying light intensities, spectral compositions, and exposure durations would also clarify their practical utility in mitigating non-visual effects.

## 6 Conclusion

It has been observed that exposure to LED light for 30 min on the prefrontal cortex can positively impact cognitive performance at the behavioral level. This effect is due to the acceleration of nonvisual senses in the higher cortical regions of the brain, leading to increased alertness. This phenomenon occurs via the ipRGC projection on the prefrontal cortex. However, it is essential to note that repetitive task performance without light exposure does not lead to increased alertness. It has also been suggested that exposure to cumulative LED light can help prolong sleep by suppressing melatonin secretion, thereby keeping alertness. The BBL (Crizal Prevencia) does not ameliorate the nonvisual senses, such as alertness against harmful blue light, during prolonged exposure to WLED for 30 min.

## CRediT authorship contribution statement

**Mousumi Ghosh:** Writing – original draft, Visualization, Software, Methodology, Investigation, Formal analysis, Data curation, Conceptualization. **Hari Prakash Palaniswamy:** Writing – review & editing, Visualization, Validation, Supervision, Resources, Project administration, Investigation, Formal analysis, Conceptualization. **Nishitha. G:** Writing – original draft, Visualization, Methodology, Investigation, Formal analysis, Data curation, Conceptualization. **Stelyna Joylin:** Writing – original draft, Methodology, Investigation, Formal analysis, Data curation, Conceptualization. **Shwetha. T.S:** Writing – review & editing, Visualization, Validation, Supervision, Resources, Project administration, Investigation, Formal analysis, Conceptualization. **M. Sanjana:** Writing – review & editing, Writing – original draft, Validation, Methodology, Investigation, Formal analysis, Data curation. **R.P. Radhika:** Writing – review & editing, Writing – original draft, Visualization, Supervision, Resources, Project administration, Methodology, Data curation.

## Disclosure statement

No potential conflict of interest was reported by the author(s).

## Data availability statement

The data supporting this study's findings are available from the corresponding author, [N.T.], upon reasonable request.

## Funding

Nil.

## Declaration of competing interest

The authors declare the following financial interests/personal relationships which may be considered as potential competing interests: Nagarajan T reports financial support, administrative support, statistical analysis, and writing assistance were provided by the 10.13039/100019305Manipal Academy of Higher Education. Nagarajan T reports a relationship with 10.13039/100019305Manipal Academy of Higher Education that includes board membership, consulting or advisory, employment, and non-financial support.

## Data Availability

The data supporting this study's findings are available from the corresponding author, [N.T.], upon reasonable request.
